# Role of Injectable Platelet-Rich Fibrin in the Management of Soft and Hard Tissue Periodontal Regeneration in Dentistry: Protocol for a Systematic Review

**DOI:** 10.2196/65137

**Published:** 2025-01-23

**Authors:** Unnati Shirbhate, Pavan Bajaj, Mayur Wanjari, Manoj Patil

**Affiliations:** 1 Department of Research and Development Sharad Pawar Dental College Datta Meghe Institute of Higher Education and Research Wardha India

**Keywords:** injectable platelet-rich fibrin, pulp regeneration, periodontal regeneration, periodontium, tissue engineering

## Abstract

**Background:**

Injectable platelet-rich fibrin (i-PRF) has the capacity to release great amounts of several growth factors, as well as to stimulate increased fibroblast migration and the expression of collagen, transforming growth factor β, and platelet-derived growth factor. Consequently, i-PRF can be used as a bioactive agent to promote periodontal tissue regeneration.

**Objective:**

We aim to compare and evaluate the effectiveness of i-PRF in periodontal tissue regeneration.

**Methods:**

We will conduct an electronic search in the following databases: PubMed, Cochrane Library, Google Scholar, Semantic Scholar, Scopus, and Web of Science. Papers will be restricted to those in English and to those that are randomized controlled trials comparing PRF or any other biomaterial with i-PRF for periodontal regeneration during dental treatment. The included papers in this review and the reference lists of pertinent reviews will be manually searched. The selection of studies, data extraction, and assessment will be carried out separately by 2 reviewers using the Risk of Bias 2 tool for the included research.

**Results:**

The success of i-PRF will be evaluated by comparing the mean difference in periodontal regeneration of soft and hard tissues in terms of gingival recession, probing pocket depth, clinical attachment level, bone gain, and gingival width. The combined effect size measurements and the associated 95% CIs will be estimated using a random-effects model. The synthesis or work for this systematic review started in October 2023 and will last until December 2025.

**Conclusions:**

i-PRF may play a role in dentistry and could enhance soft and hard tissue regeneration.

**Trial Registration:**

PROSPERO CRD42023464250; https://www.crd.york.ac.uk/prospero/display_record.php?RecordID=464250

**International Registered Report Identifier (IRRID):**

PRR1-10.2196/65137

## Introduction

### Rationale

A leucocyte-enriched platelet-rich concentrate called injectable platelet-rich fibrin (i-PRF) has recently been researched and developed to help in wound healing and increase the success of periodontal tissue regeneration [1,2]. The objective of developing an i-PRF formulation is to provide specialists with a platelet concentration in liquid form that is simple to administer and may be mixed with various biomaterials or used alone [3-6]. By adopting slower and shorter centrifugation rates, i-PRF yields significantly more regenerated cells and higher concentrations of growth factors than prior PRF formulations that used faster centrifugation speeds [7]. i-PRF has been shown to produce more fibroblast migration and have higher levels of growth factor production, as well as superior antibacterial adhesive action against a range of periodontal disease pathogens [8].

i-PRF is a novel platelet-rich concentrate that enhances periodontal tissue regeneration due to its “supraphysiological” growth factors and cell concentrations [1]. The slow release of growth factors is possible due to the dynamic fibrin liquid gel that i-PRF delivers, which embeds platelets rich in leucocytes, collagen type I, osteocalcin, and growth factors even in their liquid phase. i-PRF can improve intrinsic tissue regeneration by generating human mesenchymal stem cells (MSCs) and starting osteogenic differentiation in MSCs [9]. By allowing the inclusion of grafts without the need for additives or anticoagulants, i-PRF creates a well-agglutinated “sticky bone graft” [1].

The applicability of i-PRF as a promising regenerative adjuvant in periodontal tissue regeneration has been validated by clinical research [2]. Liquid PRF permits other alterations, like incorporating different biomaterials, in an injectable form [10]. One of the components that makes up i-PRF is fibronectin, which is an extracellular glycoprotein with a high molecular weight (approximately 440 kDa) [4,6,11]. It has been demonstrated that i-PRF centrifugation at a horizontal angle is far more effective than traditional fixed-angle centrifugation at accumulating more cells and growth factors. The physician can construct customized clotted PRF membranes (biografts) by manipulating i-PRF before clotting, which can then be combined with different regenerating agents [9].

### Objective

This systematic review will determine the effect of i-PRF in the management of soft and hard tissue periodontal defects in the context of periodontal tissue regeneration. The review will highlight the use of i-PRF as a bioactive agent capable of stimulating periodontal tissue regeneration.

## Methods

### Overview

We aim to ascertain whether i-PRF is effective in soft and hard tissue regeneration in periodontics by conducting a systematic review of the literature.

### Eligibility Criteria

We will examine eligible studies and describe how i-PRF is used for the treatment of pulp and periodontal tissue regeneration. The publications that qualify will be limited to those published in English. There will be no restrictions on publication date. We will search the identified publications’ reference lists for more papers that might meet the eligibility requirements. This review will cover the research designs of clinical randomized controlled trials (RCTs).

### Information Sources

We will search the following electronic databases for papers that fit the review’s eligibility requirements: PubMed, Cochrane Library, Google Scholar, Semantic Scholar, Scopus, and Web of Science.

### Search Strategy

First, a restricted search of MEDLINE was carried out to find applicable papers. A comprehensive search method was developed using words found in the titles, abstracts, and keywords.

### Study Selection, Data Management, and Data Collection Process

**Figure 1:**
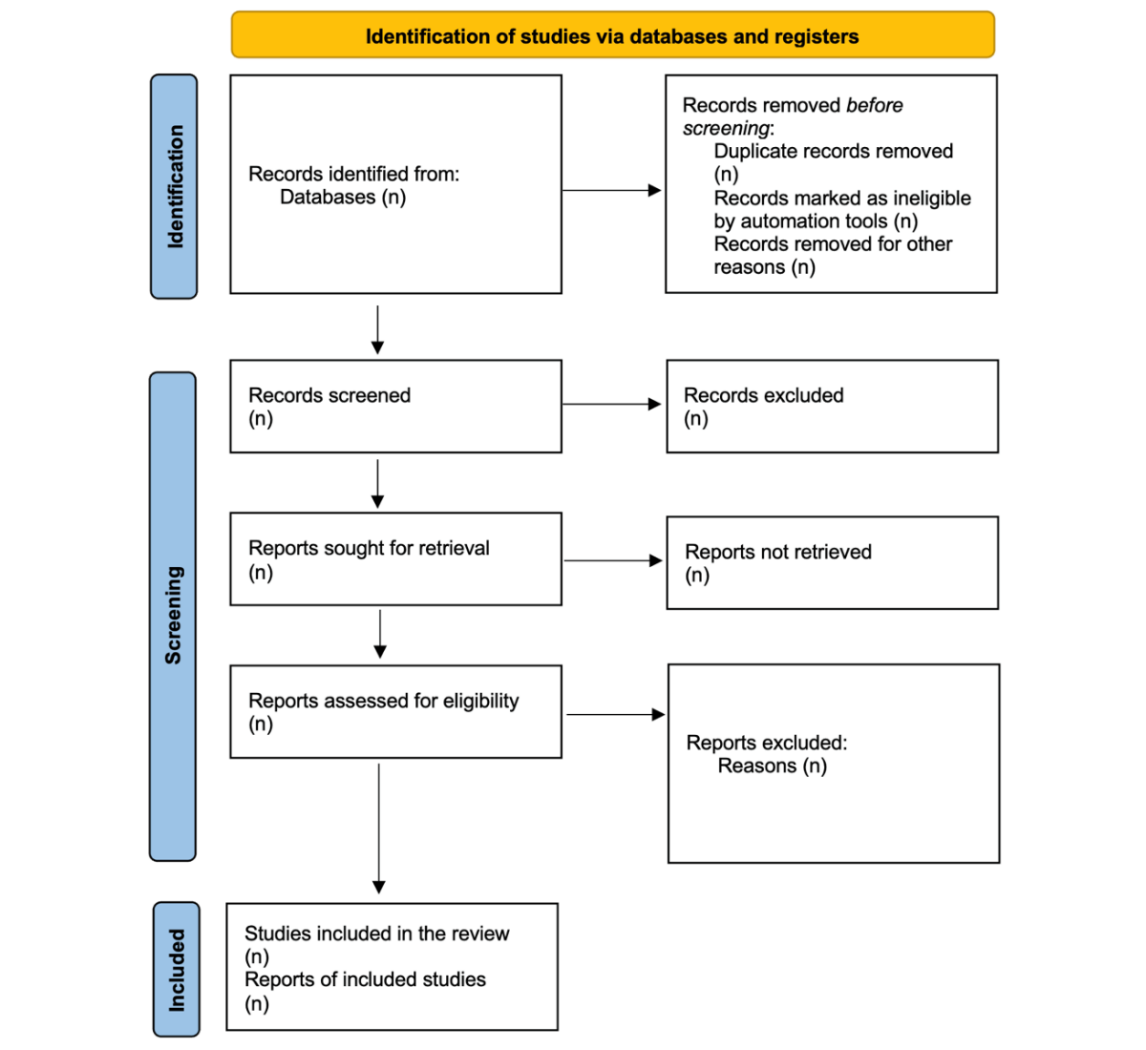
Preferred Reporting Items for Systematic Review and Meta-Analysis Protocols (PRISMA-P) flowchart.

When choosing which studies to include, the 3 reviewers (US, PB, and MW) will refer to the eligibility criteria. Records management and duplicate removal will be facilitated by the recording of selections. Blind screening of studies will be used, and differences will be settled through discussion. Two researchers will load the retrieved studies and perform title and abstract screening. Two authors will conduct a full-text review, and any disagreements will be settled by discussion or, if necessary, a third reviewer. A summary of the study selection procedure is given in Figure 1.

Reviewers will be guided by a data extraction tool applied to the included articles. Systemically healthy patients and patients receiving periodontal or pulp regenerative therapy will be included in the RCTs. Patients with a history of systemic illness, as well as pregnant women, will be excluded. A narrative summary of the results from the included research will be provided. We will assess methodological, statistical, and clinical heterogeneity. If there is enough homogeneity among the included studies, a quantitative synthesis, or meta-analysis, will be carried out.

### Data Items

Two separate, blinded reviewers (US and PB) will each record the extracted data in duplicate. The extracted data, which will include publication details, the research study’s setting, the regeneration agent, adjunctive treatment, and the defect type, will be entered into a Microsoft Excel spreadsheet. Clinical determinants will include periodontal clinical parameters, osseous defect fill, and soft tissue parameters.

### Outcomes and Prioritization

The primary outcome is to determine the role of i-PRF in soft and hard periodontal tissue regeneration in dentistry. The secondary outcomes will be whether i-PRF improves probing pocket depth reduction, gain in clinical attachment loss, and osseous fill in periodontal osseous defects.

### Risk of Bias

The Risk of Bias 2 (RoB 2) tool will be used to evaluate the risk of bias in included studies and to verify the methodological quality of the reviews. The assessments will be completed by 2 different reviewers (US and MW), with the 2 other reviewers (PB and MP) acting as arbiters to settle any disputes. The entire process will be concealed from the reviewers.

### Data Synthesis and Assessment of Heterogeneity

Statistical heterogeneity will be assessed with a random-effects model. If there is enough homogeneity among the included studies, a quantitative synthesis, or meta-analysis, will be carried out. The goal is, if at all feasible, to carry out a subgroup analysis based on the effects of various i-PRF preparation protocols on the regeneration of hard and soft tissues by varying centrifugation force, revolutions per minute, and time. Using statistical software, the results will be aggregated and computed according to the statistical guidelines cited in the most recent edition of the *Cochrane Handbook for Systematic Reviews of Interventions*.

## Results

This study is to be completed from October 2023 to December 2025. Each stage of the procedure will involve the blinding of 2 reviewers. To resolve disagreements or conflicts between the reviewers, regular team meetings will be convened. This will help to improve the review process’s transparency. Every conversation will be documented. The systematic review will be submitted for publication as soon as it is finished. The planned review has been registered with PROSPERO (CRD42023464250) and will be carried out in compliance with the guidelines provided in the Preferred Reporting Items for Systematic Review and Meta-Analysis Protocols (PRISMA-P) checklist.

## Discussion

### Anticipated Findings

i-PRF in the periodontal regenerative process offers hope for improved patient outcomes and enhanced therapeutic interventions [12,13]. It might help to provide improved healing and periodontal tissue reconstitution in the context of improvement in osseous fill or bone gain, as well as soft tissue fill, by reducing probing pocket depth and increasing clinical attachment loss. i-PRF might potentially act as a bioactive agent capable of inducing tissue regeneration. Wang et al [14] contrasted i-PRF to conventional platelet-rich plasma (PRP) and examined the osteoblast behavior of a unique therapeutic i-PRF that was 100% natural and additive-free. They found a noticeable increase in the messenger RNA levels of osteocalcin, Runx2, alkaline phosphatase, and immunofluorescent staining of osteocalcin when comparing an i-PRF group to a PRP group. The results of that study favored the use of naturally formulated i-PRF in conjunction with anticoagulants over PRP [14].

Varela et al [15] investigated the blood cell composition, morphological characteristics, type I collagen gene expression, and growth factor release of i-PRF. Blood samples from 15 people were used to create i-PRF samples. The study found that i-PRF was a potentially effective treatment option for the healing of soft and mineralized tissues because it promoted the development of a 3D fibrin network comprising platelets, leukocytes, type I collagen, osteocalcin, and growth factors. Indeed, given its flowable mixing capabilities with other biomaterials and simplified procedures, i-PRF may be used in various dental applications [15]. Iozon et al [4] investigated how human gingiva–derived mesenchymal stem cells (MSCs) proliferated and underwent osteo-differentiation in response to i-PRF; they concluded that gingiva-derived MSC proliferation was promoted by 5% i-PRF, but that an overabundance of i-PRF may hamper osteogenic induction [4].

### Strengths and Limitations

To the best of our knowledge, this is the first systematic review to focus on whether i-PRF aids in tissue regeneration. The integration of qualitative as well as quantitative data synthesis in this study will help in promoting oral and periodontal health.

Limitations of this systematic review may include variations in the study design, sample size, and methodological quality of the included studies, as well as potential bias in reporting outcomes related to the use of i-PRF for periodontal tissue regeneration. Additionally, heterogeneity in treatment protocols, variations in PRF preparation methods, and differences in follow-up duration may influence the generalizability of findings. Publication bias and the exclusion of non–English language studies could further affect the comprehensiveness of the review.

### Conclusion and Directions for Dissemination

This study aims to assess whether i-PRF has the potential for regeneration of periodontal structures. If we find that the intervention has an effect, the results will aid in tissue engineering, maintaining and promoting osseous defect gain by osseous fill, and hastening healing. Results from this study will be published in national and international academic journals. This protocol follows the PRISMA-P guidelines (Multimedia Appendix 1).

## Appendix

Multimedia Appendix 1 Preferred Reporting Items for Systematic Review and Meta-Analysis Protocols (PRISMA-P) checklist.

## Abbreviations

i-PRF: injectable platelet-rich fibrin
MSC: mesenchymal stem cell
PDGF: platelet-derived growth factor
PRISMA-P: Preferred Reporting Items for Systematic Review and Meta-Analysis Protocols
PRP: platelet-rich plasma
RCT: randomized controlled trial
RoB 2: Risk of Bias 2

## Data Availability

Data sharing is not applicable to this article as no data sets were generated or analyzed during this study.
